# Ergonomic Status of Laparoscopic Urologic Surgery: Survey Results from 241 Urologic Surgeons in China

**DOI:** 10.1371/journal.pone.0070423

**Published:** 2013-07-31

**Authors:** Boluo Liang, Lin Qi, Jinrui Yang, Zhenzhen Cao, Xiongbing Zu, Longfei Liu, Long Wang

**Affiliations:** 1 Department of Reproductive Center, Second Xiangya Hospital, Central South University, Changsha, China; 2 Department of Urology, Xiangya Hospital, Central South University, Changsha, China; 3 Department of Urology, Second Xiangya Hospital, Central South University, Changsha, China; 4 Department of Gynecologic Oncology, The Affiliated Tumor Hospital of Xiangya Medical School, Central South University, Changsha, China; Cardiff University, United Kingdom

## Abstract

**Background:**

The prolonged and frequent use of laparoscopic equipment raises ergonomic risks that may cause physical distress for surgeons. We aimed to assess the prevalence of urologic surgeons’ physical distress associated with ergonomic problems in the operating room (OR) and their awareness of the ergonomic guidelines in China.

**Methods:**

A sample of 300 laparoscopic urologists in China was assessed using a questionnaire on demographic information, ergonomic issues in the OR, musculoskeletal symptoms, and awareness of the ergonomic guidelines for the OR.

**Results:**

There were 241 survey respondents (86.7%) with valid questionnaires. Among the respondents, only 43.6% placed the operating table at pubic height during the actual operation. The majority of the respondents (63.5%) used only one monitor during the procedure. Only 29.9% placed the monitor below the eye level. More than half of the respondents (50.6%) preferred to use manual control instead of the foot pedal. Most of the respondents (95.0%) never used the body support. The respondents experienced discomfort in the following regions, in ascending order: leg (21.6%), hand (30.3%), wrist (32.8%), shoulder (33.6%), back (53.1%), and neck (58.1%). The respondents with over 250 total operations experienced less discomfort than those with less than 250 total operations. Most of the respondents (84.6%) were unaware of the ergonomic guidelines. However, almost all of the respondents (98.3%) regarded the ergonomic guidelines to be important in the OR.

**Conclusions:**

Most of the laparoscopic urologists were not aware of the ergonomic guidelines for the OR; hence, they have been suffering from varying degrees of physical discomfort caused by ergonomic issues. There is an urgent need for education regarding ergonomic guidelines in the OR for laparoscopic urologists in China.

## Introduction

With the development of minimally invasive surgery in urology, the number of laparoscopic urologic surgeries has substantially increased over the past 10 years [1]. Robot-assisted surgery has also been widely used in urology, but due to its expensive cost and the highly qualified technical level of surgeons in China and other developing countries, this alternative has been less widespread in China. Laparoscopic urologic surgery has even become the first-line surgical technique for certain urologic diseases and has several advantages, including minimal incision, little pain, short recovery time and acceptable expenses for the patients [2]. However, many surgeons develop ergonomic problems as a result of the non-neutral body postures required. These problems have led to physical complaints, such as numbness, stiffness and pain in the neck, shoulder, back and leg. Gofrit et al. [3] reported that surgeons suffer from varying degrees of physical strain during laparoscopic urologic surgery.

Ergonomics is the study of optimal designs to ensure appropriate psychological and physical interactions among the worker, product and environment [4]. The ergonomic problems of the surgeons are mainly associated with the following five issues in the operating room (OR): the design of the hand-held instrument, the height of the operating table, the number and placement of monitors, foot pedal use and body support to relieve the static posture [5]. The ergonomic guidelines for these issues have been stated in the literature to reduce the risk factors of musculoskeletal distress [6]–[10]. However, few of the guidelines in the literature have been adopted in China. To investigate the current application of these ergonomic guidelines in China and the awareness of Chinese urologic surgeons regarding the guidelines in the OR, we initiated an integrated cross-sectional study to identify probable ergonomic factors that might result in the physical complaints of laparoscopic urologists while performing the procedures.

## Methods

We performed this study during the 11th National Minimally Invasive Urology Academic Conference, which was held by the Chinese Urological Association (CUA) in Wuhan, Hubei Province on May 28–29, 2011. Our study was approved by the ethics committee of Xiangya Hospital, Central South University. The surveyed laparoscopic urologists were selected from the representatives present at the meeting. All of the surveyed urologists were screened to ensure that they specialized in laparoscopic urologic surgery and were active in clinical practice. A questionnaire was designed in which demographics, ergonomic issues in the OR, musculoskeletal symptoms and the awareness of the ergonomic guidelines were investigated. The demographic investigation included gender, age, height, practice location, the level of the hospital and the total number of laparoscopic urologic surgeries performed by the surgeon. The ergonomic issues in the OR that we investigated included the use of hand-held instruments, the height of the operating table, the number and placement of monitors, foot pedal use and body support. The musculoskeletal symptoms investigation contained a questionnaire that was elaborated upon by the ergonomics subcommittee of the Society of American Gastrointestinal and Endoscopic Surgeons (SAGE) [11], [12]. The surgeons’ awareness of the ergonomic guidelines in the OR was assessed by several questions evaluating their recognition of and attitude toward the ergonomic guidelines developed by Delft University of Technology.

The questionnaires were handed out to 300 urologic surgeons present at the meeting and were immediately returned anonymously. The data were collected and imported into a Microsoft Excel 2003 spreadsheet for a descriptive analysis using the software SPSS 18.0. The data analyses were performed using summary statistics, a chi-square test and variance analysis, and a P value<0.05 was considered statistically significant.

## Results

Three hundred questionnaires were handed out. Of these, 241 surgeons responded to the survey and completed the questionnaires with valid answers. The respondents came from hospitals in 76 cities of 18 provinces in China.

### Demographics

Table 1 illustrates the demographic information of the surgeons that responded to the questionnaire. Of the 241respondents, the average age was 44.3 years old, ranging from 28 to 61. Most of these were male (96.7%), and more than a half (50.6%) were working in hospitals with more than 1000 beds. The majority of respondents (59.8%) had performed more than 250 laparoscopic urologic surgeries.

**Table 1:** Demographic information of the respondents.

	Number(n = 241)	Percentage
Gender		
Male	233	96.7%
Female	8	3.3%
Level of hospital (number of beds)		
<500	47	19.5%
500–1000	72	29.9%
>1000	122	50.6%
Height (cm)		
<160	31	12.9%
160–170	53	22.0%
171–180	116	48.1%
>180	41	17.0%
Number of laparoscopic urologic surgeries performed in total		
0–250	97	40.2%
>250	144	59.8%

### Ergonomic Issues in the OR

#### Hand-held instruments

Most of the respondents (77.6%) used grasping and dissecting forceps with an angled ring handle, while 18.7% of the respondents used forceps with angled shank handle. Only 3.7% of the respondents used forceps with an in-line handle. Of the respondents, 53.5% used a needle holder with an in-line handle. The remaining cases used needle holders with angled shank handles (22.8%) or angled ring handles (23.7%). The majority of the respondents (53.9%) were satisfied with their grasping and dissecting forceps and needle holder.

### Operating Table Height

**Figure 1:**
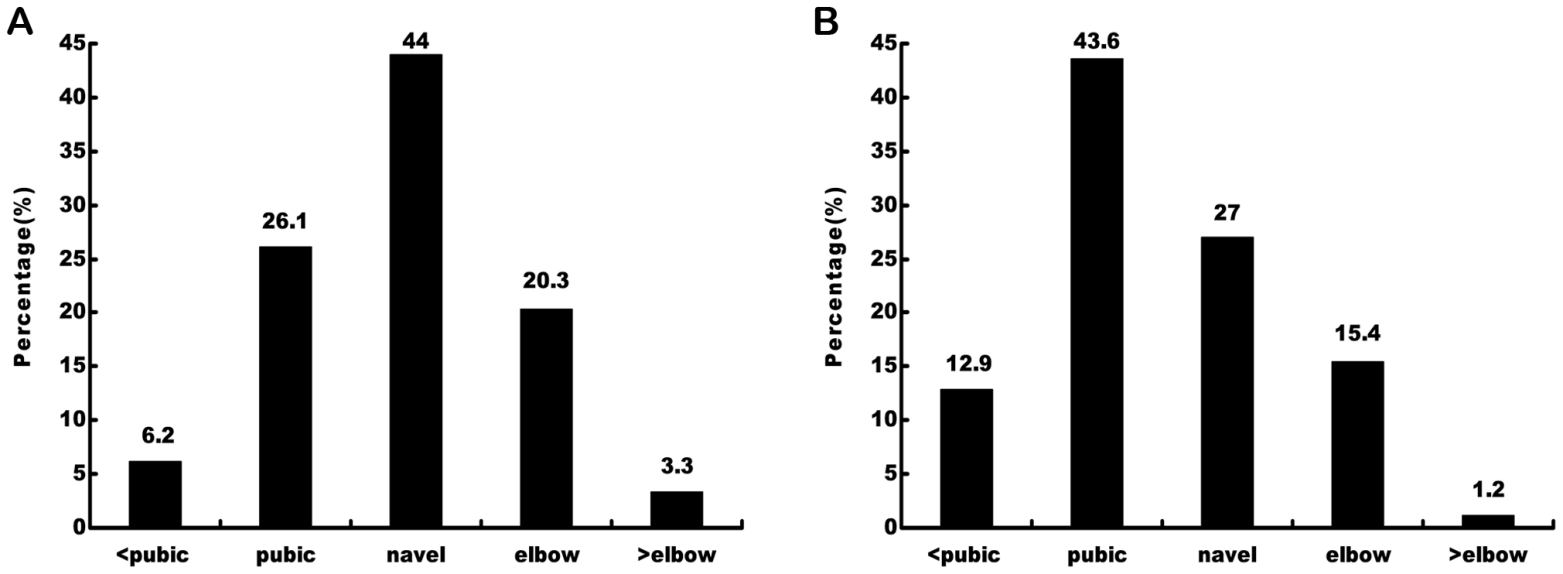
Distribution of the operating table heights used while placing the trocars (A) and during the actual laparoscopic manipulation (B).

Most of the respondents (44.0%) placed the operating table at navel height during the incision and placement of the trocars, whereas they placed the operating table primarily at pubic height (43.6%) during the actual operation (Figure 1). Among the respondents, surgeons who used the operating table at the pubic level during the actual operation experienced less discomfort in the shoulders (24/105) than those with the operating table at other heights (57/136) (P = 0.002). More than half of the respondents (64.3%) were satisfied with the height of their operating tables.

### Monitor

Most of the respondents (63.5%) used one monitor during laparoscopic urologic surgery, while the remaining respondents (36.5%) used more than one monitor to perform the procedure. The majority of the monitors (44.0%) were placed at eye level. Only 29.9% of the respondents placed the monitor below eye level. The surgeons with the monitor height below the eye level experienced less discomfort in the neck (33/72) than those with the monitor height at or above eye level (107/169) (P = 0.012). Almost 52.7% of the respondents were not satisfied with the monitors.

### Foot Pedal

Most respondents (86.3%) used either a hand control or foot pedal to activate the diathermic or ultrasonic equipment. A little more than half (50.6%) of the respondents preferred to activate the diathermic or the ultrasonic equipment using hand control. Approximately 44.8% of the respondents used more than one foot pedal, while the majority of the respondents (49.8%) used only one foot pedal during the procedure. A majority of the respondents (56.4%) were satisfied with the foot pedal. However, 43.6% of respondents who were not content with the foot pedal, for the following reasons: they often lost contact with the foot pedal; they had to look down at the foot pedal before they pushed the switch; the wrong foot pedal or switch was sometimes activated unintentionally, resulting in potential danger for the patient; and they were forced to stand on one foot while manipulating the foot pedal.

### Body Support

Most of the respondents (95.0%) never used a body support. The majority of the respondents (87%) who did use the body support found it comfortable for use.

#### Musculoskeletal symptoms

All of the respondents were asked whether they experienced symptoms in the hand, wrist, shoulder, neck, leg or back. If the answers were positive, they were also required to describe the extent of the discomfort.

**Figure 2:**
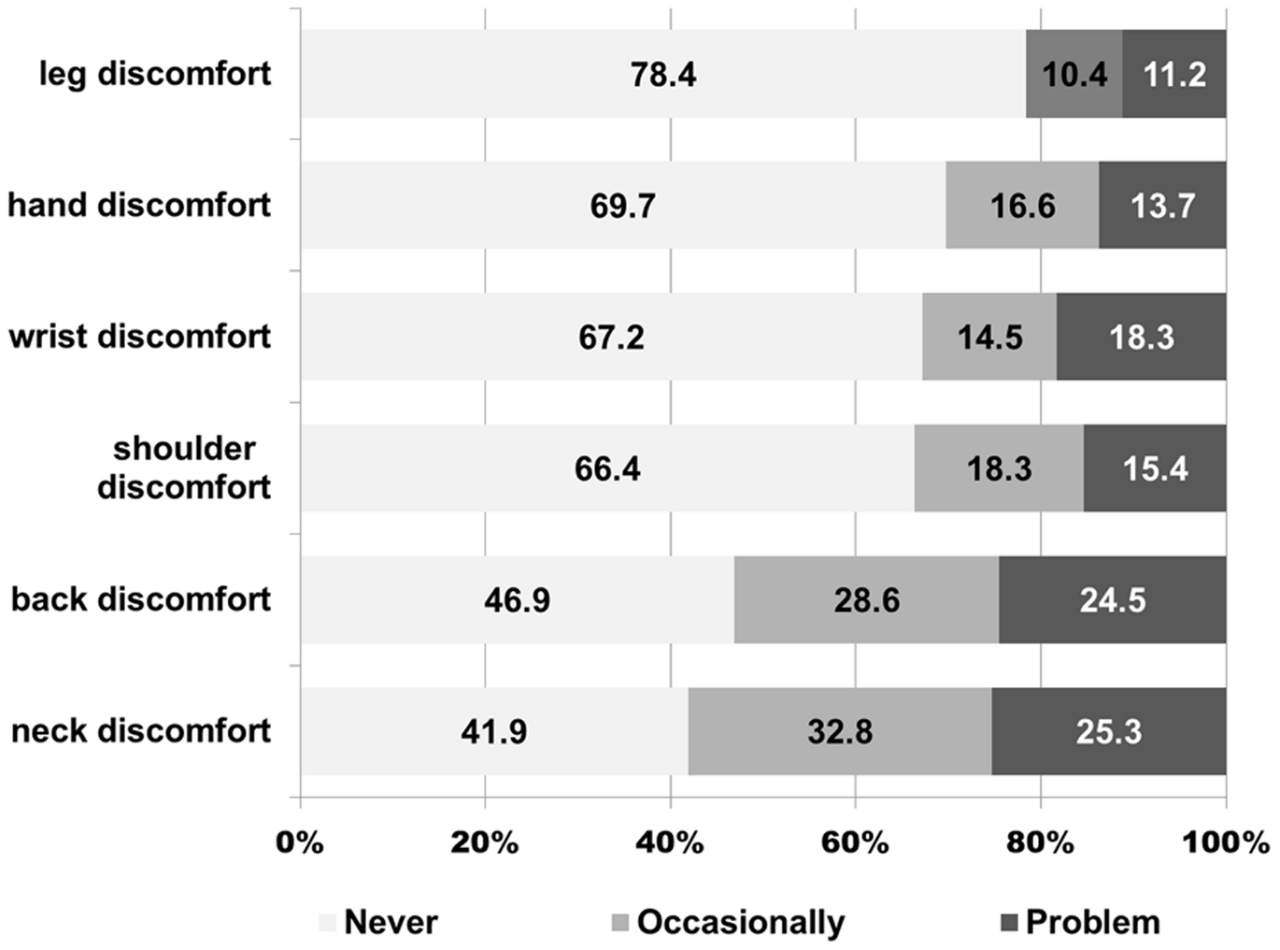
Results from the survey of musculoskeletal symptoms.

The respondents experienced the discomfort of the following parts, in ascending order: leg (21.6%), hand (30.3%), wrist (32.8%), shoulder (33.6%), back (53.1%), and neck (58.1%). (Figure 2) The respondents were divided into two groups. Group A included surgeons who had not performed more than 250 surgeries (97/241, 40.2%), and Group B contained those who had performed more than 250 surgeries (144/241, 59.8%). We found that Group A experienced more discomfort than Group B in their hands (P = 0.029), wrists (P = 0.022), and backs (P = 0.026). (Table 2).

**Table 2:** Distribution of the discomforts between Group A and Group B.

	Group A(n = 97)	Group B (n = 144)	P value
Hand discomfort	37 (38.1%)	36 (25.0%)	0.029
Wrist discomfort	40 (41.2%)	39 (27.1%)	0.022
Shoulder discomfort	35 (36.1%)	46 (31.9%)	0.505
Neck discomfort	59 (60.8%)	81 (56.3%)	0.480
Leg discomfort	21 (21.6%)	31 (21.5%)	0.982
Back discomfort	60 (61.8%)	68 (47.2%)	0.027

Group A = respondents who had previously performed no more than 250 laparoscopic surgeries.

Group B = respondents who had previously performed more than 250 laparoscopic surgeries.

*P<0.05* was considered statistically significant.

#### Guidelines

Only 2.9% of the respondents were aware of the ergonomic guidelines concerning hand-held instruments, operating table height, placement of the monitor, foot pedal use and the application of body support. Most of the respondents (94.6%) never received any training or education on ergonomic guidelines in the OR. However, almost all of the respondents (98.8%) stated that ergonomic guidelines in the OR are important and should be considered.

## Discussion

With the rapid development of minimally invasive surgery, laparoscopic urologic surgery has quickly matured, but the increasing use of this technique has been accompanied by ergonomic problems. Majeed et al. [13] reported a case of occupational injury in a laparoscopic surgeon in 1993. They attributed this injury to the non-ergonomic design of the hand-held instrument. As the complaints grew, an increasing number of researchers have focused on ergonomic problems in the OR. In 2001–2003, van VMA et al. [5], [6], [8], [14], in the faculty of industrial design engineering at Delft University of Technology, gradually developed a series of guidelines for surgeons to reduce the risk of laparoscopic injury. Nevertheless, more and more cases of occupational injury among laparoscopic surgeons have been reported. However, there is little in the literature on the ergonomic situation of the laparoscopic urologists in China. As far as we know, this is the first nationwide survey of the ergonomic perceptions among Chinese laparoscopic urologists. The response rate of our study is 80.3%, which is much higher than those in comparable studies of minimally invasive surgeons of other countries [15]–[18]. The respondents in our study represent hospitals in 76 cities of 18 provinces in China. Based on the relatively high number of respondents to our survey and its status as a nationwide survey covering almost all of the administrative regions in China, we deemed our data to be representative of the current perception of Chinese urologic surgeons toward ergonomic issues in the OR.

The complaints of the surgeons decreased with increased laparoscopic experience. This result is consistent with the observations of Sari V et al. [19] and Hemal et al. [20] among laparoscopic surgeons. In these studies, surgeons with laparoscopic surgical experience of less than two years showed more discomfort. Uhrich ML et al. [21] found that less experienced surgeons have higher electromyography amplitudes than experienced surgeons, which is a sign of higher muscle tension that may result in musculoskeletal symptoms. Experienced laparoscopic urologists were better able to manipulate the tools and had a greater awareness of the risk factors in the OR; thus, they were able to avoid being injured during operations.

Placement of the monitor is one of the most important ergonomic factors in the OR. The guidelines [7] for monitor placement suggest that the monitor be placed in front of each surgeon to avoid axial rotation of the spine in the horizontal plane and approximately 10–15° below eye level to avoid neck extension in the sagittal plane. Most of our respondents did not place the monitor below the eye level; thus, neck discomfort was the most prominent problem among the surgeons, presumably due to the extended duration of posture that included bending their necks forward. Because the patient is situated in the lateral trendelenburg or reverse-trendelenburg position for retroperitoneal approaches in China, the operating surgeon and assistant must stand on either side of the patient during the laparoscopic urologic surgery. The majority of the respondents (63.5%) used only one monitor, while the assistant had to rotate his neck and spine to gaze at the monitor that was placed next to him. In these conditions, the surgeon places more stress on his neck and back.

The operating table height in our study represents the height of the operating surface during the procedure. Most of the respondents performed the incision and placement of the trocars with the operating surface height at navel level, which is the ideal height for open surgery. Nonetheless, a majority of the respondents placed the operating surface at levels other than pubic level, which was suggested in the ergonomic guidelines. Van Veelen et al. [22] suggested that the optimum height for laparoscopic surgery should be located at a factor of 0.7–0.8 of the length from the floor to the elbow height of the surgeons. To eliminate the inconvenience of measuring the elbow height of every surgeon during practice, the optimum operating surface height was suggested to be pubic level. At that height, the surgeon could maintain a neutral posture in which the laparoscopic instruments could be placed close to elbow level of the surgeons to minimize joint excursion and discomfort in the arms and shoulders while performing the precise actions required by the procedures. However, in China, laparoscopic urologic surgery is always performed on patients in the lateral trendelenburg or reverse-trendelenburg position; thus, the operating surface is higher than that of normal laparoscopic surgery. The operating table cannot be lowered to the proper height while maintaining the operating surface at pubic level. The surgeon would need to work on one side, rotate his/her spine and raise his/her extremities to manipulate the instruments, and a greater muscular load would be placed on the surgeon’s back, shoulder, and upper extremities.

The use of hand-held instruments influences the surgeons’ alignment of the hands and wrists. In our study, most of the surgeons used the laparoscopic forceps with an angled ring handle to grasp and dissect, and needle holders with in-line positioned shank handles were most commonly used. These results agree with those of a survey of European laparoscopic surgeons by van VMA et al. [15]. The forceps fitted with an angled ring handle prevent rotation of the lower arm, but greater pressure is placed on the fingers. Potential nerve damage could result in hand discomfort, such as finger numbness and stiffness. Instruments fitted with an angled shank handle, which were used by the remainder of respondents, are used to relieve this hand discomfort, but they resulted in a loss of sensitivity in the fingers [23]. The needle holders with an in-line positioned shank handle were also at risk of injuring the extreme ulnar deviations of the wrist during the suturing process. As technology has developed, the design of these instruments has improved greatly with respect to ergonomics. However, the newly designed instruments remain unable to completely solve the ergonomic problems in the upper extremities.

Although fewer respondents complained about the foot pedal and body support, many deficiencies in their design also exist. More than half of the respondents preferred to activate the diathermic or the ultrasonic equipment with their hands rather than their feet. Van VMA et al. [24] designed novel types of foot pedals to improve these ergonomic aspects so that the surgeon would experience less discomfort in using the foot pedal. Apparently, these have not yet become widely used in China. The body support system has also been rarely applied in China. Almost all of the respondents had never used any type of body support during laparoscopic urologic surgeries. However, the majority of the respondents who had used the body supports regarded them to be beneficial in the relief of body fatigue and stress on the back. In previous studies, Albayrak A et al. [25] found that the body support was effective in reducing the muscle activity in the leg muscles and in relieving the strain on the back and shoulders.

Most of our respondents were unaware of the ergonomic guidelines and had never received any specific training or education related to them. Bagrodia et al. [26] showed in his study that 25% of the surveyed surgeons were affected by physical discomforts or complaints and would fully consider ergonomic problems when deciding on an operative approach. Xiao DJ et al. [27] found that a skills lab with surgical simulators could help surgeons to learn about the ergonomic factors in the OR through a series of lessons. Almost all of the respondents in our study realized the importance of ergonomic issues during the laparoscopic urologic procedure and that better ergonomic conditions would lead to better performance and less discomfort. There is an urgent need to popularize relevant knowledge concerning ergonomics in the OR among laparoscopic urologists in China. Directing the attention of these surgeons toward ergonomic issues in the OR through training before the actual procedure should ameliorate their musculoskeletal strain. Meanwhile, manufacturers should provide more ergonomic instruments and devices to improve the ergonomic conditions of the OR.

Admittedly, our study has certain limitations. First, the questions on physical discomfort were subjectively answered by the respondents. The respondents’ answers to the questionnaire represent self-reported perceptions that might provide a more idealized picture of the situation than actually exists. Thus, our results are less indicative than those of studies using more objective indexes. Nevertheless, many additional measures were present in or out of the OR that were not addressed by the questionnaire, such as the air conditioning, lighting, flooring, operation type, average operating time, workload and so on. Furthermore, fewer surgeons have experience with laparoscopic urologic surgery compared to those who practice conventional open urologic surgery, despite its increasingly widespread application; this limits the number of surgeons who could participate in this survey. Finally, the ergonomic problems in the OR require further research to define the relationships among all of these ergonomic factors in or out of the OR. In the authors’ view, these problems could all be improved in the near future by guiding the attention of surgeons toward ergonomic factors and the development of these medical techniques.

In conclusion, ergonomic problems in the OR during laparoscopic urologic surgery have gradually been reported in recent years. The present ergonomic situation of laparoscopic urologists in China is not optimal. Most of the laparoscopic urologists in China have been suffering discomfort to varying extents from not following the guidelines of the five main issues in the OR. There is urgent need for us to improve this situation, not only by focusing on this isolated issue, but by considering all of the ergonomic issues simultaneously. On the one hand, we should demand improvement in the ergonomic designs of the products in the OR. On the other hand, there is urgent need to popularize the principles of ergonomics and to simulate operational training using these ergonomic guidelines in China.
